# In or out?—Suggested criteria to systematically offer different treatment options to patients with body dysmorphic disorder

**DOI:** 10.3389/fmed.2022.986781

**Published:** 2022-12-15

**Authors:** Christian M. G. Stierle, Klaus-Michael Taube

**Affiliations:** ^1^Psychology School, Fresenius University of Applied Sciences, Hamburg, Germany; ^2^Dermatological Practice Dr. Zarzour, Halle, Germany

**Keywords:** body dysmorphic disorder, psychotherapy, inpatient treatment, outpatient treatment, differential indication

## Introduction

Body dysmorphic disorder (BDD) is a highly complex mental disorder, which is characterized by preoccupation with one more perceived flaw in the individual’s appearance. It often results in repetitive behavior strategies to hide, check, or alter these flaws causing massive distress and impairment as well as low quality of life (1). Recent studies confirm that prevalence among dermatological patients is higher than in the general public (2) with 10.5 vs. 2.1% (3), suggesting that especially patients suffering from hyperhidrosis, alopecia, and vitiligo are vulnerable to BDD. Patients often worry about facial issues such as skin and hair, which are omnipresent to others. It often goes along with high degrees of external shame as well as internal shame, fearing not to fulfill one’s individual standards and ideals. BDD is often associated with a wide range of comorbidities such as depression, social anxiety, obsessive compulsive disorder (OCD), and substance use disorder. Besides these, there are comorbid personality disorders such as avoidant personality disorder (4). BDD results in poorer social adjustment, relationships, problems, and occupational functioning (5). Very concerning aspects of BDD are high rates of suicidality (6) and self-manipulation, such as skin-picking behavior and self-mutilation. These post a special challenge on treatment and patient and physician/therapist relationship.

## Psychotherapeutic treatment

There is sound evidence that cognitive behavioral therapy (CBT), besides pharmacological therapy with high-dose selective serotonin reuptake inhibitors (SSRIs), is the first line of treatment showing good effects in symptom reduction (7). Besides these promising results, there is rather limited data on long-term therapy effects (8), suggesting that many patients stay symptomatic and still show risk factors. There has been little research so far on which treatment setting is most suitable and profitable for patients (9). This is partly due to a lack of specialized treatment options such as outpatient centers and specialized clinics. Nevertheless, many aspects of severe BDD symptoms suggest that solely outpatient counseling or therapy might sometimes not be enough to properly tackle BDD and that therapy requires a structured decision process. In addition to outpatient therapy, specialized inpatient therapy can provide a more intense and secure therapy process including group therapies together with other BDD patients, movement and art therapy as well as the possibility to incorporate further medical investigations and pharmacological co-treatment. Furthermore, it can also provide a helpful therapeutic community helping to address shame and reduce social withdrawal. As many BDD patients see dermatologists first before possibly moving on to psychotherapy, dermatologists often obtain a special role and responsibility in evaluating which kind of treatment might be most suitable for their patients. Possible screening tools for the dermatological practice are the Body Dysmorphic Disorder Questionnaire (BDDQ) (2) or its dermatological version BDDQ-DV, as well as the Dysmorphic Concern Questionnaire (DCQ) (10).

## Criteria for in- and outpatient treatment

**FIGURE 1:**
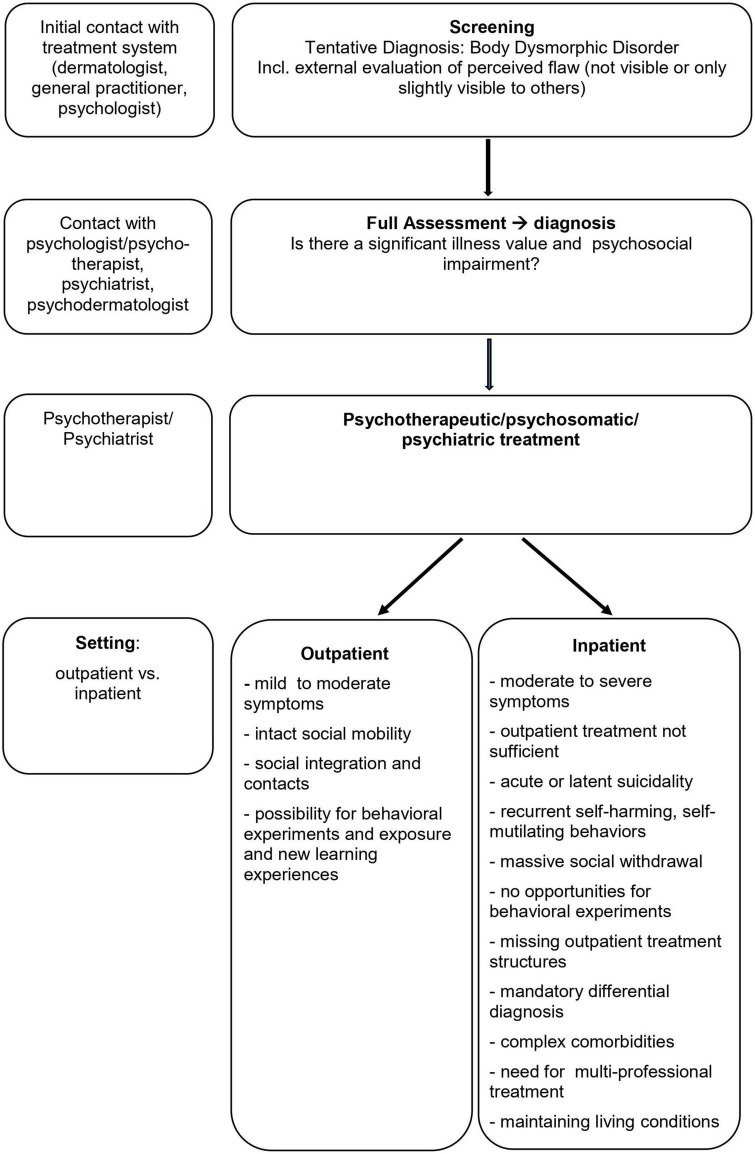
Criteria for outpatient vs. inpatient treatment.

The following model (Figure 1) incorporates the first criteria that could be helpful in making the decision and whether in- or outpatient psychotherapy would be preferable and describes a possible process of how to come to this decision.

Besides relying solely on symptom severity it might be helpful to take a closer look at aspects that facilitate or hinder therapy processes. Massive social avoidance and isolation may be a factor that enormously obstructs outpatient treatment, leading to a situation where core treatment elements such as behavioral experiments and exposure as well as mirror confrontations, etc., may not be possible. Furthermore, complex symptomatology and complex comorbidities (e.g., personality disorders) that require specific differential diagnosis or a multi-professional treatment approach (e.g., dermatological treatment next to psychotherapeutic) suggest an inpatient setting. In cases of frequent self-harm or suicidal ideation, a more secure and stabilizing therapy setting seems appropriate. Finally, living conditions that help to maintain BDD symptoms such as high family accommodation or ongoing bullying or criticism urge the removal of the patient from these surroundings and place them in a more constructive environment.

We highly recommend taking the inpatient setting into consideration, as it provides the chance for an intensive and multi-professional treatment approach and offers patients the chance to encounter other BDD patients which have proven to be very useful in our clinical experience. Specific and intensive therapy might help to save resources and lower treatment costs in the long run and prevent patients and healthcare professionals from recurring unsuccessful outpatient therapies.

Overall, this requires more specialized treatment settings with specialized centers, both out- and inpatient, which focus on body dysmorphic disorders, incorporating psychological, psychiatric, and psychodermatological competencies, as well as close cooperation between dermatologists, psychologists, and psychiatrists and a thorough screening and assessment process.

## Author contributions

CS conceptualized and wrote the manuscript and developed the suggestions for differential criteria on in- and outpatient treatment. K-MT substantially contributed ideas and reflection on the manuscript and added ideas on clinical implications. Both authors contributed to the article and approved the submitted version.
